# Surface visualisation of bacterial biofilms using neutral atom microscopy

**DOI:** 10.1111/jmi.70038

**Published:** 2025-10-03

**Authors:** Nick A. von Jeinsen, David J. Ward, Matthew Bergin, Sam M. Lambrick, David M. Williamson, Richard M Langford, Lisa F. Dawson, Vibhuti Rana, Sushma Shivaswamy, Xuening Zhou, Michelle Mikesh, Vernita D. Gordon, Brendan W. Wren, Katherine A. Brown, Paul C. Dastoor

**Affiliations:** ^1^ Department of Physics Cavendish Laboratory University of Cambridge Cambridge UK; ^2^ Centre for Organic Electronics University of Newcastle Callaghan New South Wales Australia; ^3^ Department of Infection Biology London School of Hygiene and Tropical Medicine London UK; ^4^ XBiotech USA Inc. Austin Texas USA; ^5^ Center for Nonlinear Dynamics The University of Texas at Austin Austin Texas USA; ^6^ Interdisciplinary Life Sciences Graduate Program The University of Texas at Austin Austin Texas USA; ^7^ Department of Physics The University of Texas at Austin Austin Texas USA; ^8^ LaMontagne Center for Infectious Disease The University of Texas at Austin Austin Texas USA; ^9^ Center for Biomedical Research Support The University of Texas at Austin Austin Texas USA; ^10^ The Oden Institute for Computational Engineering and Sciences The University of Texas at Austin Austin Texas USA

**Keywords:** bacterial biofilms, scanning helium microscope

## Abstract

The scanning helium microscope (SHeM) is a new technology that uses a beam of neutral helium atoms to image surfaces non‐destructively and with extreme surface sensitivity. Here, we present the application of the SHeM to image bacterial biofilms. We demonstrate that the SHeM uniquely and natively visualises the surface of the extracellular polymeric substance matrix in the absence of contrast agents and dyes and without inducing radiative damage.

## LAY DESCRIPTION

Neutral atom microscopy (NAM) is a new field that uses beams of neutral atoms to image surfaces. The first NAM instrument, the Scanning Helium Microscope (SHeM), uses a particle beam of neutral helium atoms to form the image, rather than photons (light) or electrons. By exploiting the large scattering cross‐section of helium to atomic scale surface features, coupled with its neutral/inert nature and extremely low energy (∼60 meV), the SHeM is a uniquely sensitive imaging technology capable of visualising surfaces entirely non‐destructively. The SHeM enables the study of materials (and their applications) whose visualisation is constrained, whether by intrinsic insensitivity, the requirement for surface treatments/coatings, or by surface damage altering the material under investigation. Here we present the SHeM imaging of bacterial biofilms, highlighting its sensitivity to the surface of extracellular polymeric substance matrix in the absence of contrast agents and dyes and without inducing radiative damage.

## 1 INTRODUCTION

Bacterial biofilms are complex multicellular aggregates that form on virtually any surface across diverse ecosystems,^1^ playing critical roles in environmental processes^2^ and infectious diseases.^3^ Formation of these biofilms involves the production of an extracellular polymeric substance (EPS) matrix that is typically composed of a variety of materials including exopolysaccharides, proteins, lipids, and extracellular DNA (eDNA).^4^ The biofilm surface creates a protective interface between an external environment and an internal supportive microenvironment that enables bacteria to survive and thrive.^5^

Traditional imaging techniques, such as optical and electron microscopy, have provided valuable insights into biofilm architecture but are often limited by their inability to differentiate between surface and subsurface features.^6^ This constraint can be a result of the thin and transparent nature of the biofilm surface, as well as the sample preparation techniques used for imaging that can create structural artefacts due to losses of biofilm matrix components.^7^ The scanning helium microscope (SHeM) is a new technique that offers an alternative modality for surface imaging. It utilises neutral helium atoms with extremely low energy (∼60 meV) and short wavelength (∼0.05 nm) to interact solely with the outermost surface layers of a sample with no effective radiative damage.^8^ This extreme surface sensitivity arises because helium atoms scatter from the outermost electron distributions of the surface,^9^ enabling the visualisation of delicate structures. In addition, the natural contrast mechanisms inherent to this method enables samples to be visualised without the need for dyes, stains, or metal coatings.^10^

In this study, the SHeM has been applied to image biofilm surfaces. We demonstrate the utility of this imaging technique using biofilms prepared from the major nosocomial pathogen *Clostridioides difficile*. Comparative SHeM images of native biofilm and biofilm treated with DNase I (an enzyme that disrupts the EPS matrix) are presented. By contrasting SHeM images with optical and SEM micrographs, we illustrate how SHeM complements traditional imaging techniques. It provides a unique visualisation of how biofilm surface can completely encase bacteria as well as evidence of how the surface itself can be disrupted by the degradation of a key component, eDNA in this example.

## 2 METHODS

### 2.1 Biofilm preparation

Biofilms of *C. difficile* standard strain 630 were cultivated on Thermanox™ (Thermo Fisher Scientific) coverslips as previously described.^11^ Briefly, the bacteria were grown in brain heart infusion (BHI) broth supplemented with 0.5% yeast extract and incubated anaerobically at 37°C for 48 h to form mature biofilms. For EPS disruption, biofilms were treated with 10 µg/mL DNase I (Sigma‐Aldrich) for 15 min at room temperature to degrade eDNA within the matrix.^11^ A common concern when imaging biological samples under high vacuum, as required for SEM and SHeM, is the potential for dehydration or collapse of hydrated EPS matrices, which may lead to structural artefacts. In this demonstration, samples were chemically fixed prior to imaging, with 4% paraformaldehyde for 15 min and washed with phosphate‐buffered saline (PBS), which minimises gross morphological changes, but may not preserve all aspects of the native hydrated structure. Three independent biological replicates per condition were prepared and multiple regions imaged per replicate; images shown are representative.

### 2.2 Optical microscopy

Images were captured using an Olympus BH‐2 Microscope fitted with Olympus Neo SPlan lenses (5×, 20×, 50×). Normarski interference contrast enhancement is used to highlight any variation in surface topography. Micrographs are recorded using a Nikon D7000 camera. The comparative images were captured using a Keyence VHX‐7000 microscope with micrographs recorded using an integrated 4K CMOS image sensor.

### 2.3 Confocal microscopy

Biofilms were stained with 0.1 µM BacLight (TM) Red (Molecular Probes cat# B‐35001) and 10 µM Hoechst 33342 (Thermo Fisher Scientific cat #62249). Images were captured using a Nikon W1 spinning disk confocal microscope with a water 60× objective. On the vertical axis, *z*‐stack images were acquired at a step size of 0.3 µm. The field of view was 135 × 135 µm.

### 2.4 Scanning electron microscopy (SEM)

High‐resolution SEM images shown in Figures 2 and 3 were acquired using a Hitachi SU8600 Scanning Electron Microscope (SEM), a field‐emission SEM equipped with a high‐brightness cold field emission (CFE) gun. Optimal contrast in the micrographs was achieved by using an acceleration voltage of 1 kV and emission current of 20 mA, selected to maximise secondary electron (SE) emission while minimising sample damage. Best contrast was achieved using the Lower Detector (LD), positioned below the objective lens and the working distance was maintained at approximately 2 mm to optimise SE collection efficiency. Images were recorded at a resolution of 1280 × 960 pixels.

SEM images shown in Figure S5 were prepared using enhanced biofilm imaging methods.^7^ *C. difficile* cells, cultured for 72 h on Thermanox slides, were fixed overnight using 2% glutaraldehyde (Electron Microscopy Sciences, Hatfield, PA), 0.1% alcian blue (Sigma, St. Louis, MO), in 0.1 M sodium cacodylate buffer at pH 7.4 (Electron Microscopy Sciences, Hatfield, PA).  The next day, samples were gently washed three times in 0.1 M sodium cacodylate buffer, then postfixed and stained in 1% osmium tetroxide with 1% tannic acid in 0.1 M sodium cacodylate buffer. After 2 h, the samples were gradually dehydrated using 30%, 60%, 75%, 90%, 95% and absolute ethanol (Thermo Fisher, Waltham, MA) and hexamethyldisilazane (HMDS, Ted Pella, Redding, CA), and then air dried. Samples were mounted to stubs with carbon tape, then coated with 8 nm of iridium using a Cressington 208HR sputter coater (Ted Pella, Redding, CA). SEM imaging (Zeiss Gemini 2 460 Scanning electron microscope) was conducted with an InLens detector and a 4 kV accelerating voltage (Center for Biomedical Research Support Microscopy and Sauer Structural Biology Lab at UT Austin; RRID:SCR_022951).

### 2.5 Scanning helium microscopy (SHeM)

The biofilms grown on the Thermanox™ coverslips were mounted in the scanning helium microscope (SHeM) sample chamber under high vacuum (10^−8^ mbar), without any additional coatings or treatments.

The SHeM employs a novel imaging technique where a beam of neutral helium atoms is directed at the sample surface.^12^ The helium atoms, with an energy of ∼63 meV, scatter on the sample surface upon interaction.^13^ The exclusively surface‐scattering nature of helium at the ultra‐low energies used in the SHeM ensures that every material is effectively opaque to the helium beam.^14^ This property allows for high‐resolution imaging of surface topography without penetrating the material, providing true surface visualisation.^15^ Consequently, the SHeM is exquisitely sensitive to topographical features at the atomic to nanometre scale, and to variations in composition that affect surface electronic structure and roughness. Other contrast forming mechanisms have been observed, originating in the fine detail of the helium scattering distribution, however these effects are very small compared to topographic changes and thus are generally only distinguishable on atomically smooth surfaces. Since the helium atoms do not carry a charge, the sample must be mechanically scanned under the stationary beam to form an image, pixel by pixel, which contrasts with charged‐particle microscopies where beam scanning is standard.^16^ Thus, the SHeM obviates the need for sample preparation like staining or coating and the imaging process preserves the native integrity of the surface features observed. All images were taken using a pixel size of 0.25 µm at a dwell time of 1.8 s per pixel.

### 2.6 Haralick image analysis

Texture analysis was conducted using the Haralick method. Grey‐level co‐occurrence matrices (GLCMs) were generated using MATLAB's GRAYCOMATRIX function, applied to greyscale images and computed at four directional offsets (0°, 45°, 90°, and 135°) to capture orientation‐dependent texture characteristics. The matrices were normalised so that each entry represented probability values rather than raw frequency counts. Haralick texture features were extracted using MATLAB's GRAYCOPROPS function, including contrast (quantifying intensity variation between neighbouring pixels), correlation (measuring linear dependency between pixel intensities), energy (representing uniformity through the sum of squared GLCM entries), homogeneity (assessing local similarity of pixel intensities), and entropy (capturing randomness in intensity distribution, computed separately). A sliding window approach was employed to segment the image and compute Haralick parameters at localised regions, enabling spatially resolved texture analysis. These features were used to classify and compare texture patterns across image samples.

## 3 RESULTS AND DISCUSSION

This study is presented as a demonstration of the capability of SHeM for biofilm imaging, rather than as a statistically powered biological investigation. For each condition (untreated and DNase I‐treated biofilms), three independent biofilms were prepared and imaged across multiple regions per sample. The images shown are representative of the observed trends across replicates. Our focus is on illustrating the unique surface sensitivity and imaging modality provided by SHeM to support its further adoption in biofilm research.

**FIGURE 1:**
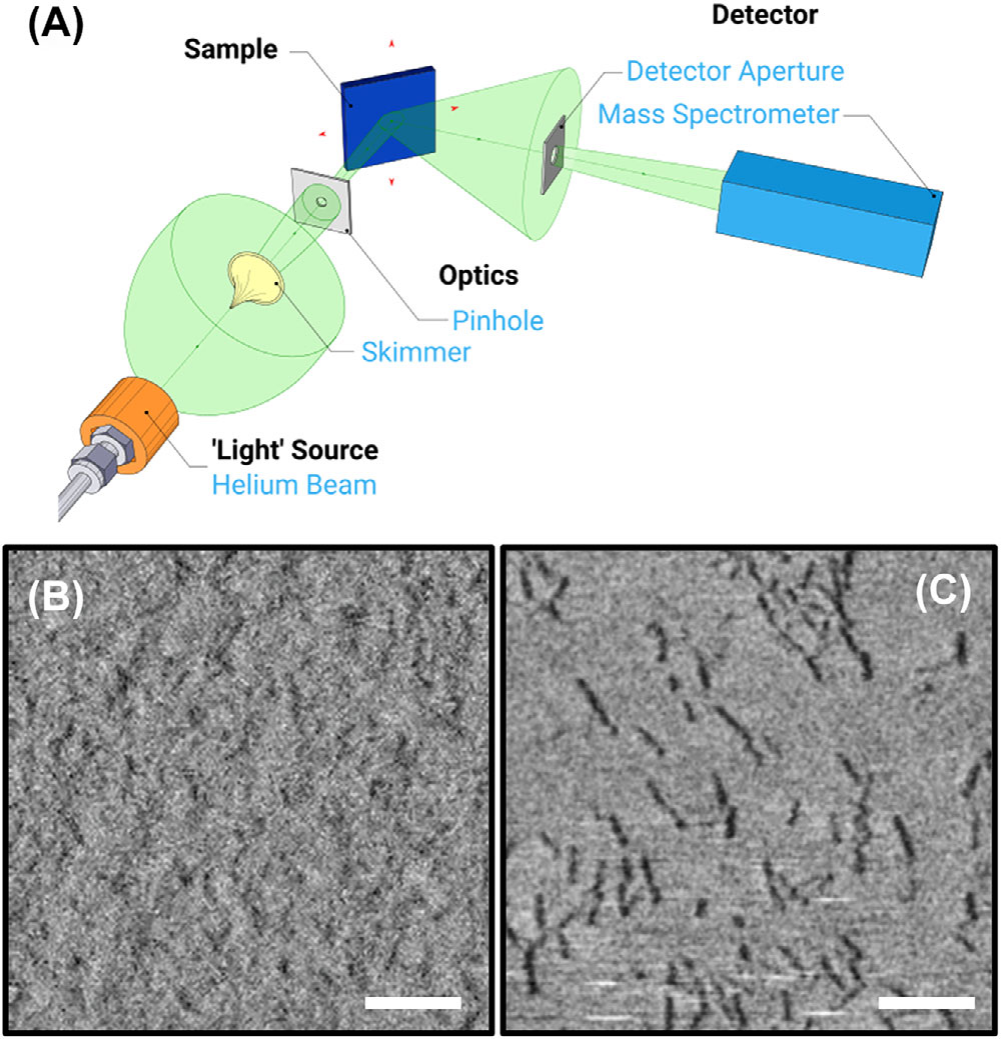
(A) Schematic of the SHeM optical arrangement.^19^ (B) SHeM micrograph of native late‐growth *C. difficile* biofilm surface. (C) SHeM micrograph of DNase I‐treated late‐growth *C. difficile* biofilm surface. Scale bars are 10 µm.

The SHeM setup (Figure 1a) consists of a high‐pressure (∼70 bar) helium source generating a near‐monochromatic helium atom beam through supersonic expansion into a low‐pressure (∼10^−3^ mbar) chamber. The beam passes through a 100‐µm skimmer aperture and a 470 nm pinhole before interacting with the sample surface. Scattered helium atoms are collected through a 1‐mm detector aperture and detected by a custom‐built mass spectrometer.^17^ The lateral resolution of the instrument is estimated from the beam width to be ∼300 nm, which is set primarily by the width of the incident helium beam and the size of the detection aperture. While this provides true surface sensitivity, it imposes limits on the smallest lateral features that can be resolved, particularly when investigating biofilm EPS matrices, which may present features below this scale. However, recent work has demonstrated that SHeM can reveal topographical variations in ultrathin organic films well below 100 nm thickness, exploiting the sensitivity of helium atom scattering to subtle surface structure and composition.^18^ Thus, while the lateral resolution is on the order of hundreds of nanometres, the SHeM is capable of detecting surface changes due to the presence or removal of thin EPS overlayers, even when these are not directly resolvable as distinct features.

Figure 1b shows the SHeM micrograph of a late‐growth untreated *C. difficile* biofilm. The image appears featureless, indicative of an EPS matrix that completely covers the bacterial cells and obscures underlying structures, consistent with the protective role of the EPS in shielding bacteria from external threats. This extreme surface sensitivity of SHeM contrasts with optical microscopy, where the EPS is largely transparent, and the underlying bacteria are visible. In contrast, Figure 1c displays the SHeM micrograph of a biofilm treated with DNase I. Here, distinct bacterial cells are visible, indicating that the enzymatic degradation of eDNA has disrupted the EPS matrix and exposed the bacteria.

**FIGURE 2:**
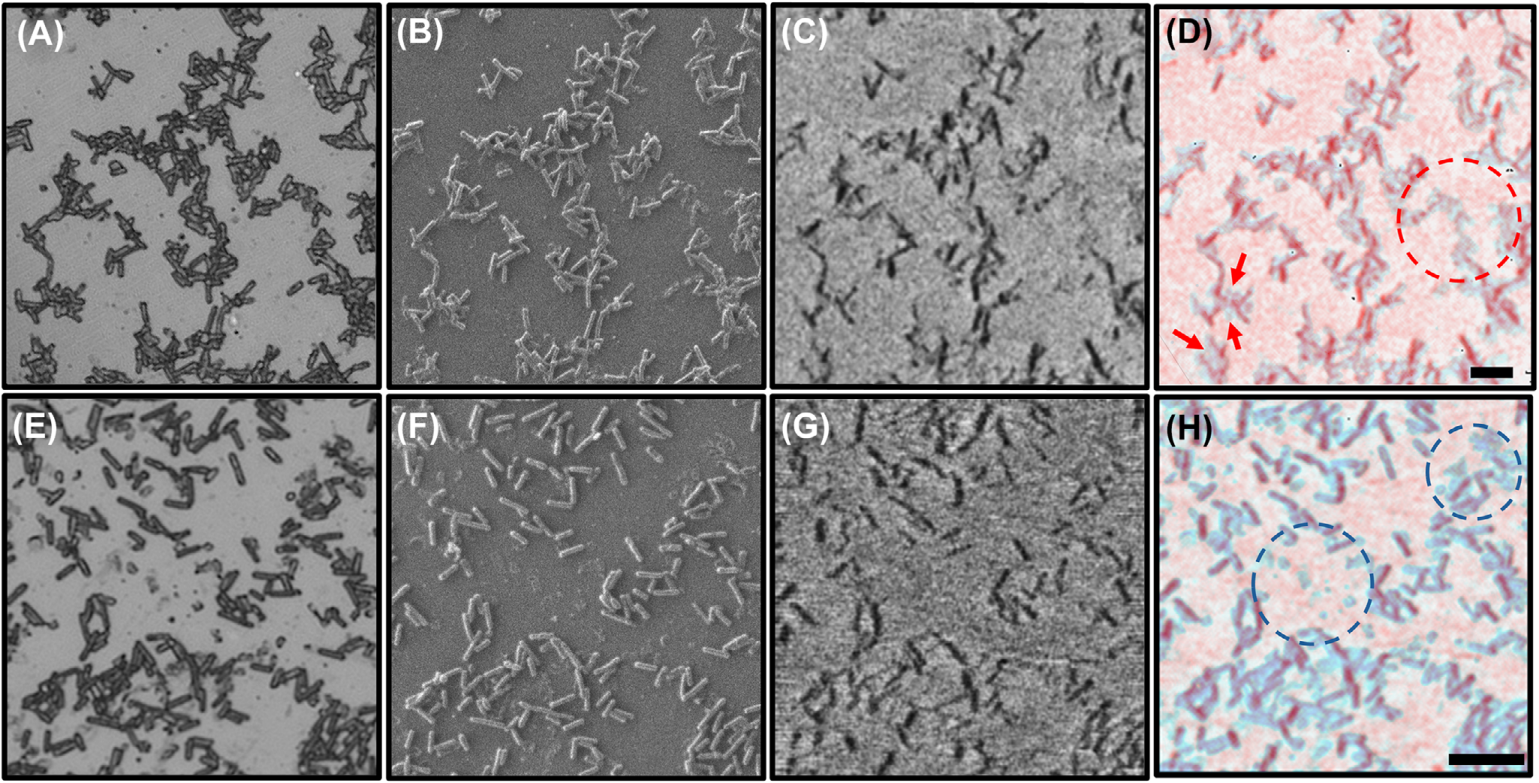
(A) Optical micrograph, (B) SEM micrograph, (C) SHeM micrograph and (D) SHeM (red) – optical (blue) micrograph overlay of the native *C. difficile* biofilm. (E) Optical micrograph, (F) SEM micrograph, (G) SHeM micrograph and (H) SHeM (red) – optical (blue) micrograph overlay of the DNase I‐treated *C. difficile* biofilm. The red arrows and dashed circle highlight crevice and interconnected regions, respectively, that are visible in the optical image but not in the SHeM micrograph of the native *C. difficile* biofilm. The blue dashed circles highlight interconnected regions that are visible in the optical image but not in the SHeM micrograph of the DNase I‐treated *C. difficile* biofilm. The scale bars are 10 µm.

Figure 2 shows the comparison of the optical, SEM and SHeM micrographs of the lower‐density biofilm edge region for the untreated and treated biofilm surfaces. In general, all three imaging techniques reveal that the untreated sample shows clusters of more interconnected bacteria, while the enzyme‐treated sample displays more isolated cells and fragments of disrupted biofilm. In addition, there are several structural features (for both the untreated and treated surfaces) that are present in the optical and SEM micrographs that appear to be obscured in the SHeM micrograph.

To highlight these masked structural features, Figure 2 also shows the SHeM (red)–optical (blue) overlay micrographs for both the native and DNase I‐treated *C. difficile* biofilms, with similar behaviour observed for the SHeM–SEM overlays. For the untreated biofilm (Figure 2d), the regions that are visible in the optical/SEM image but not in the SHeM micrograph correspond to areas with crevice‐like features (red arrows) and larger interconnected regions (red dashed circle). For the treated biofilm (Figure 2h), the overlay highlights residual fragments (blue dashed circles) that are apparent in the optical/SEM image but are obscured in the SHeM image, suggesting that after enzyme treatment, some biofilm components remain and affect helium atom scattering.

The structure and composition of native and enzyme‐treated *C. difficile* biofilms is well understood, with the distribution of key components (such as eDNA, protein and lipids) studied using SEM and confocal laser‐scanning microscopy with specific fluorescent stains. This previous work by Dawson et al. revealed that the biofilms consist of an EPS‐matrix (containing eDNA as well as cell surface and intracellular proteins) that forms a protective layer around the bacteria.^11^

Optical and electron microscopy are relatively insensitive to the non‐electron dense EPS overlayer and thus visualise primarily the underlying cells.^7^, ^20^ By contrast, helium atom microscopy is highly surface sensitive and consequently is strongly scattered by the EPS‐matrix. As such, it seems reasonable to hypothesise that the obscured features in the SHeM micrographs compared to the optical/SEM images arise from the presence of EPS‐matrix.

To further confirm this hypothesis, Haralick image analysis was undertaken on the optical, SEM and SHeM micrographs. This approach to image analysis quantifies the spatial relationships between pixel intensities in an image to extract statistical features such as contrast (measuring intensity variation between neighbouring pixels), correlation (evaluating relationships between pixel values across an image), energy (representing texture uniformity) and homogeneity (assessing local similarity of pixel values).^21^

Table 1 shows the Haralick texture analysis of the *C. difficile* biofilm across optical, SEM, and SHeM imaging modalities. A key observation is the increase in contrast and decrease in energy across all imaging techniques, consistent with the enzyme treatment inducing greater intensity variations and disrupting textural uniformity. The optical and SHeM imaging shows the most pronounced reduction in energy (from 0.391 to 0.242, and 0.162 to 0.115, respectively) and increase in contrast (from 0.037 to 0.102, and 0.663 to 0.747, respectively) indicating macroscopic biofilm degradation and a loss of uniform texture. In comparison, the SEM micrographs exhibit homogeneity values that are constant within error, and only slight shifts in the other parameters, indicating that SEM is relatively insensitive to the surface structural changes induced by the enzyme treatment.

**TABLE 1:** Haralick parameters calculated for optical, SEM and SHeM micrographs of the native and DNase I‐treated biofilm surfaces. The mean and standard deviation of the contrast, correlation, energy and homogeneity parameters calculated from the grey‐level co‐occurrence matrix computed at the four directional offsets (0°, 45°, 90°, and 135°) are presented.

*C. Diff* biofilm	Haralick parameter	Optical	SEM	SHeM
untreated	Contrast	0.037 ± 0.007	0.207 ± 0.038	0.663 ± 0.173
	Correlation	0.977 ± 0.005	0.818 ± 0.034	0.670 ± 0.090
	Energy	0.391± 0.003	0.313 ± 0.019	0.162 ± 0.014
	Homogeneity	0.981 ± 0.004	0.897 ± 0.018	0.777 ± 0.033
treated	Contrast	0.102 ± 0.020	0.214 ± 0.036	0.747 ± 0.176
	Correlation	0.964 ± 0.007	0.819 ± 0.030	0.631 ± 0.088
	Energy	0.242 ± 0.008	0.302 ± 0.017	0.115 ± 0.011
	Homogeneity	0.949 ± 0.010	0.894 ± 0.018	0.732 ± 0.034

While the averaged Haralick analysis (across the three imaging modes) is consistent with the expected overall disruption in biofilm surface texture post‐DNase I treatment, it does not provide any spatially resolved texture analysis. However, this analysis can be achieved by using a sliding window to compute the Haralick parameters at every pixel thus enabling a comparison of texture patterns across the optical, SEM and SHeM micrographs.

**FIGURE 3:**
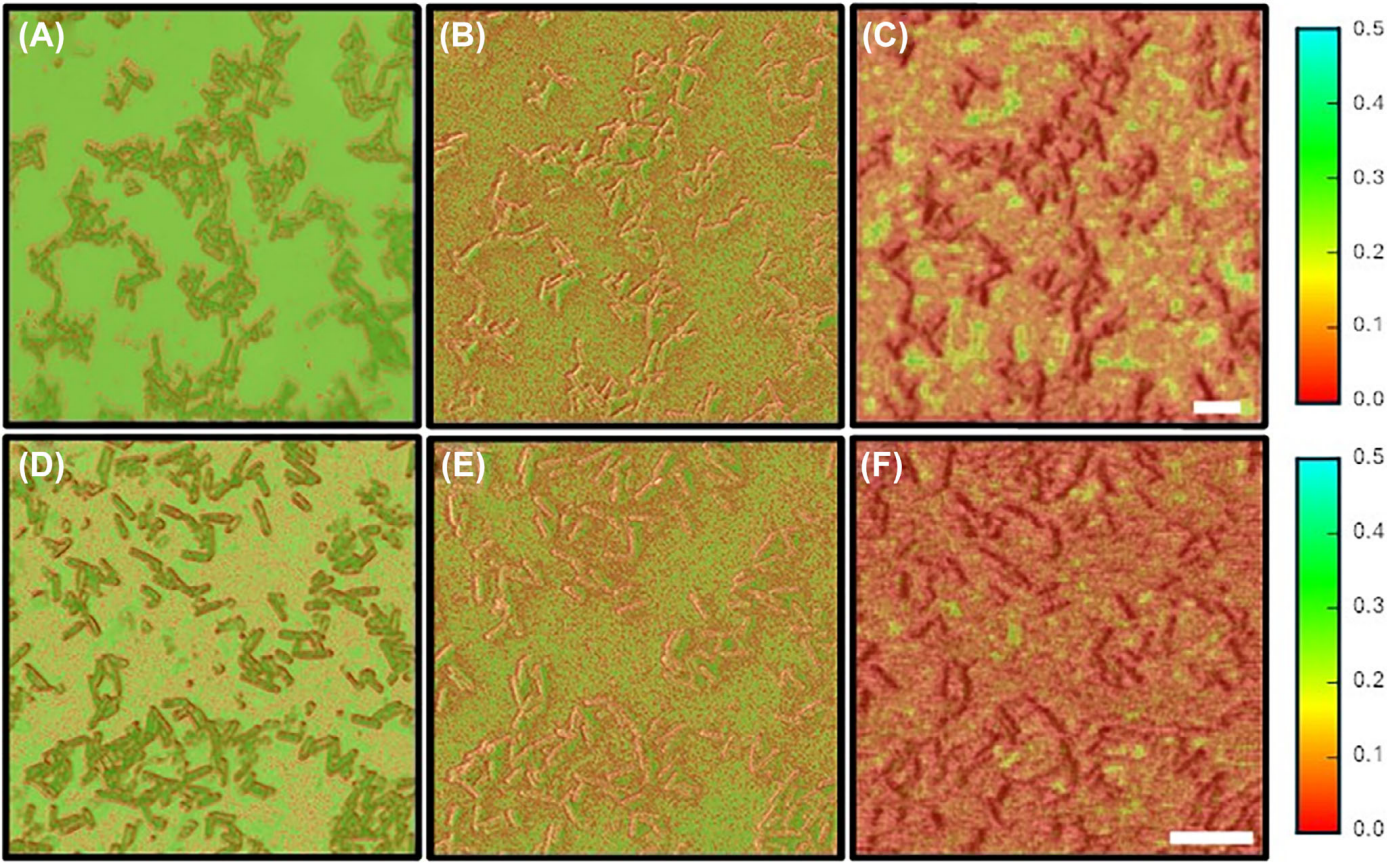
(A) Optical (grey) – Haralick Energy parameter (red‐green) overlay micrograph; (B) SEM (grey) – Haralick Energy parameter (red‐green) overlay micrograph; (C) SHeM (grey) – Haralick Energy parameter (red‐green) overlay micrograph of the native *C. difficile* biofilm; (D) Optical (grey) – Haralick Energy parameter (red‐green) overlay micrograph; (E) SEM (grey) – Haralick Energy parameter (red‐green) overlay micrograph; (F) SHeM (grey) – Haralick Energy parameter (red‐green) overlay micrograph of the DNase I‐treated *C. difficile* biofilm. The scale bars are 10 µm. The colourbar shows the mean Haralick Energy parameter colour contrast scale.

Figure 3 shows the spatial distribution of the Energy Haralick parameter overlaid on the optical, SEM and SHeM micrographs for the native and DNase I‐treated *C. difficile* biofilms. The textural uniformity decreases with enzyme treatment across all imaging modes, consistent with the trends observed in the values shown in Table 1. In addition, a clear trend in the spatial distribution of the surface texture observed by the three imaging modes can be seen. For the native biofilm surface, the SHeM overlay exhibits a structure that is distinct from the SEM and optical overlays. In particular, Figure 3c shows contiguous regions of intermediate (orange) surface texture that lie between the bacterial clusters, especially across the crevice‐like features and interconnected regions identified in Figure 2d. By contrast, the optical image shows regions of high (green) surface texture that is uniform between the bacterial clusters, whereas the SEM image has a more random intervening surface texture. Moving to the enzyme treated surfaces, the optical and SEM images exhibit an increasingly randomised surface texture between bacterial clusters. Again, the SHeM overlay of the enzyme‐treated biofilm (Figure 3f) is distinctly different, with the intermediate surface texture appearing to spread across more of the surface and again especially across the regions highlighted in Figure 2h.

The spatial distribution of the other Haralick parameters (contrast, correlation and homogeneity) was also computed (see Figure S1). However, these maps did not show the distinctly characteristic structures seen in the Energy maps. Moreover, injecting even a relatively small amount of noise into the images makes little apparent difference to the image but results in a complete loss of textural uniformity (see Figures S2 and S3). As such, it is unlikely that the observed differences in texture are a consequence of variations in signal:noise between the three imaging modalities.

In comparison, Figure S4 shows confocal images of the individual bacteria embedded as well as a 3D reconstruction of the biofilm (thickness ∼20 µm). This image is similar to previously published images by Dawson et al.^11^ SEM images of these native and DNase I treated *C. difficile* biofilms clearly show a degradation of the matrix consistent with the images in Figures 1, 2, 3. However, the outer surface of the biofilm is too thin and transparent to visualise^7^, ^20^ using either confocal or SEM techniques.

The discrepancies between SHeM and optical/SEM texture image overlays highlight the complementary nature of these techniques. While optical and electron microscopy penetrate the biofilm and visualise the underlying cells, the SHeM's sensitivity to sub‐nanoscale changes in surface topography and composition^18^ makes it particularly sensitive to the presence of the EPS‐matrix. Indeed, the untreated biofilm's SHeM texture image overlay shows a network consistent with a contiguous EPS‐covered surface that partially obscures some of the surface features observed in the optical and SEM images. Whereas the enzyme‐treated biofilm's SHeM texture image overlay shows a more homogenous surface layer, most likely residual biofilm components that continue to scatter the incident helium beam and thus obscure parts of the surface.

While this work demonstrates the SHeM's sensitivity to surface topography (especially in low‐density biofilms), under certain conditions EPS upper layer structures can be observed with conventional imaging techniques. For example, treating dense bacterial macrocolony biofilms with OsO_4_ heavy metal fixatives and subsequent coating with heavy metal Au/Pd layers can deliver sufficient contrast for the matrix layer to be observed in low energy SEM.^22^, ^23^ Although beyond the scope of this study, future work optimising the obtained SEM images, by modifying either sample processing and/or the imaging parameters, might facilitate the visualisation of the thin EPS matrix on the bacterial surface. However, in circumstances where the EPS is inaccessible to conventional imaging, the SHeM technique provides important complementary information.

As with any new technique, there are limitations. The relatively low throughput of the SHeM and its requirement for vacuum‐compatible, fixed samples currently limit the number of specimens that can be processed. In addition, while SHeM offers exceptional surface sensitivity, the ability to separate different contrast mechanisms (topography vs. composition) remains an area of ongoing research. Dehydration during vacuum imaging is an inherent challenge for hydrated biological specimens, and thus the degree to which the observed features correspond to the native hydrated EPS remains an open question. However, as the technique matures, improvements in sample preparation and imaging throughput are expected. Indeed, SHeM can be compared to quantitative phase imaging (QPI), which has transitioned from a technology‐development‐driven to an application‐focused field.^24^ QPI encompasses a family of interference‐based optical techniques that can provide label‐free imaging of transparent biological cells reconstructed into three‐dimensional holograms using instruments such as the HoloMonitor®.^25^ QPI can determine cell and organelle morphologies using phase tomography, while also quantifying cell density to enable the probing of physiological processes in living cells, such as intracellular transport. However, as with all optical techniques, complex scattering effects (such as absorption and multiple scattering) can complicate analysis and yield image artefacts,^26^ as compared to SHeM where surface kinematic scattering is dominant. While QPI systems such as HoloMonitor® provide powerful three‐dimensional reconstructions of transparent samples, SHeM offers unparalleled specificity for the outermost surface, making the two techniques complementary.

This study demonstrates that the SHeM provides a uniquely sensitive approach to imaging biofilms with a sensitivity to the overlying EPS‐matrix. The SHeM is particularly sensitive to the presence of an overlying EPS layer, as confirmed by both the loss of surface detail in SHeM images compared to SEM/optical and the quantitative increase in Haralick contrast. This observation is consistent with the established composition of these biofilms,^11^ in which the EPS forms a contiguous surface layer that can obscure bacterial morphology in surface‐scattering modalities. This capability is significant since the EPS plays a crucial role in biofilm stability, antimicrobial resistance, and interaction with the environment. Indeed, the SHeM's non‐destructive, label‐free imaging of the outermost surface layer is particularly well suited to studying EPS dynamics, antimicrobial treatments, and surface‐bacterial interactions that are inaccessible by conventional microscopy. This approach can enhance our understanding of biofilm formation, maturation, and response to treatments, providing valuable information for developing strategies to combat biofilm‐associated infections. Beyond biofilms, SHeM has potential for a wide range of soft matter, polymer, and biomaterial surfaces where surface structure is critical but conventional probes fail. Future work could include time‐resolved studies of surface changes, correlative imaging with chemical probes, and the development of in situ environmental SHeM imaging to address the challenges of hydrated or living samples.

## 4 CONCLUSION

In summary, we have demonstrated the first use of neutral atom microscopy to image bacterial biofilms. The SHeM is a new technical development whose extreme surface sensitivity delivers complementary imaging to traditional techniques such as optical and electron microscopy. The technique's unique contrast enables measurement of the EPS matrix on sparse biofilm surfaces, providing insight into biofilm architecture and the effects of enzymatic treatments such as DNase I. Consequently, the SHeM has the potential to become a valuable tool in microbiology for studying biofilm surfaces and interactions without altering native structures. Ongoing development of environmental SHeM and correlative imaging workflows will further enhance its value in microbiology.

## AUTHOR CONTRIBUTIONS

N.A.v.J., D.J.W., and D.M.W. performed the optical microscopy, SHeM imaging and data analysis. N.A.v.J., R.M.L., and M.M. performed the electron microscopy. L.F.D. and V.R. prepared the biofilm samples. X.Z. performed the confocal microscopy. K.A.B., B.W.W., V.D.G., and S.S. contributed to the interpretation of results and manuscript preparation. P.C.D. conceived the study, supervised the project, and wrote the manuscript with input from all authors.

## CONFLICT OF INTEREST STATEMENT

The authors declare no conflicts of interest.

## Supporting information



Supporting Information

## Data Availability

The datasets generated and analysed during the current study are available from the corresponding author on reasonable request.
