# Supplementary material for: Surface visualisation of bacterial biofilms using neutral atom microscopy
Source: J Microsc. 2025 Oct 3;301(1):107–15. doi: 10.1111/jmi.70038 (PMC12746369; doi:10.1111/jmi.70038)
Supplement: Supplementary file 1 — Supporting Information [file JMI-301-107-s001.docx]

Surface Visualization of Bacterial Biofilms using Neutral Atom Microscopy

**Authors:**

Nick A. von Jeinsen^1^ David J. Ward^1^, Matthew Bergin^2^, Sam M. Lambrick^1^, David M. Williamson^1^, Richard M Langford^1^, Lisa F. Dawson^3^, Vibhuti Rana^4^, Sushma Shivaswamy^4^, Xuening Zhao^5,6^, Michelle Mikesh^7^, Vernita D. Gordon^5,6,8,9^, Brendan W. Wren^3^, Katherine A. Brown^1,10^, and Paul C. Dastoor^1,2^

**Supporting Information**

**
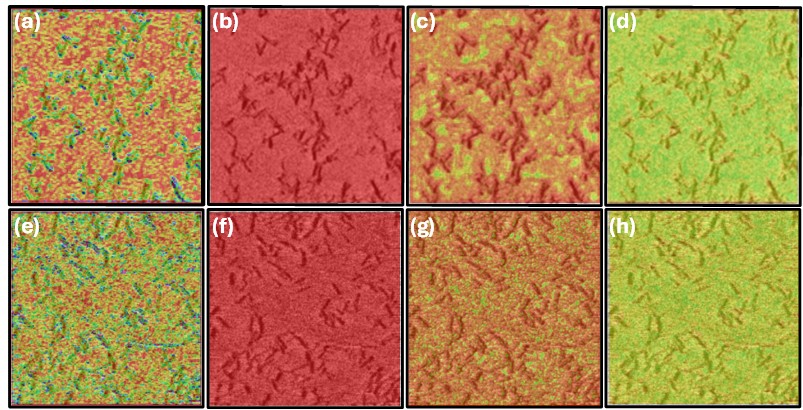
**

Figure S1:

SHeM (grey) – Haralick parameter (red-green) overlay micrographs for the native C. difficile biofilm. (a) Contrast, (b) Correlation, (c) Energy, (d) Homogeneity.

SHeM (grey) – Haralick parameter (red-green) overlay micrographs for the DNase I-treated C. difficile biofilm. (e) Contrast, (f) Correlation, (g) Energy, (h) Homogeneity.

**
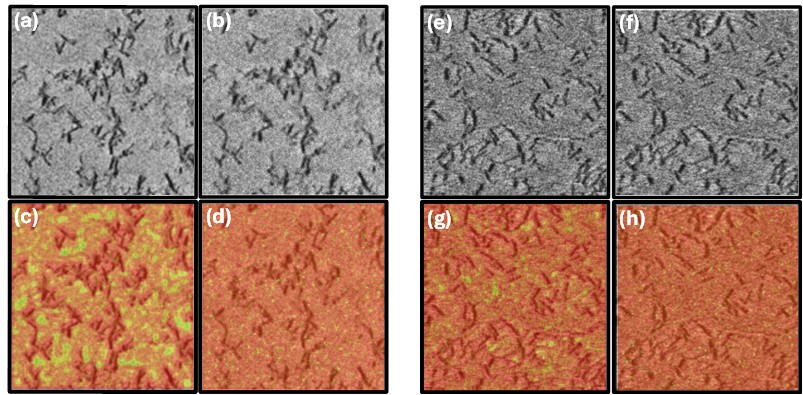
**

Figure S2:

Effect of injected noise on SHeM imaging of the native C. difficile biofilm. (a) SHeM micrograph, (b) SHeM micrograph with 1% Gaussian white noise, (c) SHeM (grey) – Haralick Energy parameter (red-green) overlay micrograph, (d) SHeM with 1% Gaussian white noise (grey) – Haralick Energy parameter (red-green) overlay micrograph.

Effect of injected noise on SHeM imaging of the DNase I-treated C. difficile biofilm. (e) SHeM micrograph, (f) SHeM micrograph with 1% Gaussian white noise, (g) SHeM (grey) – Haralick Energy parameter (red-green) overlay micrograph, (h) SHeM with 1% Gaussian white noise (grey) – Haralick Energy parameter (red-green) overlay micrograph.

Figure S3:

Effect of injected noise on SEM imaging of the native C. difficile biofilm. (a) SEM micrograph, (b) SEM micrograph with 1% Gaussian white noise, (c) SEM (grey) – Haralick Energy parameter (red-green) overlay micrograph, (d) SEM with 1% Gaussian white noise (grey) – Haralick Energy parameter (red-green) overlay micrograph.

Effect of injected noise on SEM imaging of the DNase I-treated C. difficile biofilm. (e) SEM micrograph, (f) SEM micrograph with 1% Gaussian white noise, (g) SEM (grey) – Haralick Energy parameter (red-green) overlay micrograph, (h) SEM with 1% Gaussian white noise (grey) – Haralick Energy parameter (red-green) overlay micrograph.

Figure S4:

Confocal images of native C. difficile biofilms imaged with a 60 times water objective magnification (a) representative Z-stack image showing bacteria embedded within the biofilm. The scalebar is 10 microns, (b) 3D reconstruction the biofilm showing a thickness of ~20 microns, (c) 3D reconstruction of a media-only sample showing a lack of surface features (control).

Figure S5:

SEM images of native and DNase I-treated C. difficile biofilms, respectively at 500 times (a and d, scalebar is 20 microns), 2 000 times (b and e, scalebar is 5 microns) and 10 000 times (c and f, scalebar is 1 micron) magnification. Comparison of images at 500 times and 2 000 times magnification show the effect of DNase I treatment on the matrix of the biofilm. Images at 10 000 times magnification show the presence of bacteria (rods) and matrix components in both samples.
